# WORK-RELATED OPPORTUNITY COSTS OF PROVIDING UNPAID FAMILY CARE

**DOI:** 10.1093/geroni/igz038.491

**Published:** 2019-11-08

**Authors:** Stipica Mudrazija

**Affiliations:** Urban Institute, Washington, District of Columbia, United States

## Abstract

Older Americans living in the community who need help with basic activities of daily living overwhelmingly rely on unpaid care provided most commonly by working-age family members. Because unpaid family care limits the demand for nursing facilities and reduces expenses paid by Medicaid and other government programs, previous estimates of its economic value have mostly focused on estimating the benefits of unpaid family care. However, to assess accurately the overall economic value of unpaid family care and define better the scope for policy intervention, it is also important to account for the costs of such care, yet our knowledge of their magnitude remains limited. This study assesses the impact of unpaid family caregiving on the likelihood of working and hours worked for caregivers, and calculates the related cost of forgone earnings today and in 2050. To do so, it matches family caregivers from the National Study of Caregiving with non-caregivers from the Panel Study of Income Dynamics, and uses projections from the Urban Institute’s DYNASIM microsimulation model to inform calculations of future costs of foregone earnings. Results suggest that the cost of foregone earnings attributable to caregiving is currently about $67 billion. By mid-century, it will likely more than double, outpacing the growth of disabled older population as the share of better-educated caregivers with higher earning capacity increases. Policymakers can use these results to inform their current and future policy efforts aimed at assisting family caregivers who are facing the challenge of balancing work and caregiving responsibilities.

